# Supplementary material for: Learning curve and surgical outcome of robotic assisted colorectal surgery with ERAS program
Source: Sci Rep. 2022 Nov 29;12:20566. doi: 10.1038/s41598-022-24665-w (PMC9709162; doi:10.1038/s41598-022-24665-w)
Supplement: Supplementary file 1 — Supplementary Information 1. [file 41598_2022_24665_MOESM1_ESM.zip › LSC Raw data-Ñ[▒K/148/O_ñw╝╨░O▒Kñσ.pdf]

正式摘要(電子簽章：00000233752416)

W52 / W52

臺中榮民總醫院

Operation Note

[redacted]

- 手術日期：2022/01/06
- 手術醫師：蔣鋒帆，Fellow 楊曙榮，VS 江世偉，R 林志安，R 蔡濬樟
- Anesthesia : GE
- Position : Supine

Diagnosis

- Pre-operative diagnosis : CECAL CA WITH HIGH CEA RO LUNG METS
- Post-operative diagnosis : 1. Cecal ca, cT3NxMO s/p laparoscopic right hemicolectomy on 2022/1/6
  2. Left ureteral stone s/p laparoscopic removal of ureteral stone s/p D-J insertion on 2022/1/6

Surgical Indication

- To removal primary tumor

Operation

- 1. Laparoscopic right hemicolectomy with side to end anastmosis by EEA 29mm (small bowel end to T colon side) + GIA 75mm-blue *1
  2. Ileo-colic artery and middle colic right branch was ligated via Hemo-clips.
  3. Take down of hepatic flexure : (+)
  4. Cut margin: proximal (ileum) >10 cm, (colon) >10 cm
  5. Repair of mesentery defect : Nil.
  6. we consult GU doctor, left ureteral stone was remove under laparoscope and left D-J was inserted

Finding

- 1. A 4*4 cm central ulcerative tumor over cecum area, cT3 with suspected enlarge LN
  2. No other gross tumor was found.
  3. There was no visible liver metastasis or visible peritoneal seeding.
  4. No obvious ascities

Procedure

- 1. General endotracheal anesthesia was induced and then the patient was placed in the supine position.
  2. Preoperative antibiotics were given. A Foley catheter and nasogastric tube were placed. The abdomen was prepped and draped in standard sterile fashion.
  3. A small vertical incision was made left to the umbilicus and a 12mm trocar was inserted into the peritoneal cavity by open method.
  4. Pneumoperitoneum was established with CO2 inflation. Laparoscope was inserted and then a 12mm trocar were inserted over LUQ of abdomen.
  5. Another 5mm trocar was inserted over LLQ of abdomen ; The other 5mm trochar was inserted over RLQ of abdomen.
  6. The small bowel was inspected and retracted to downward by grasper. The peritoneum overlying the mesentery was then scored with electrocautery and the ileocolic artery was identified, ligated with endoclips, and transected via Harmonic scalpel.
  7. The remaining vessels to the cecum were also divided and ligated. Using electrocautery and Harmonic scalpel, omentum was freed from the transverse colon and lesser sac was entered.
  8. The middle colic artery and middle colic artery right branch were all further ligated via Hemo-clips and Harmonic scalpel.

- 9. After the vessel were all under controlled, the colon was freed from its peritoneal attachments along the line of Toldt from the cecum to the hepatic flexure.
  10. Hemostasis was checked in the operative field. Cecum, ascending colon and hepatic flexure were mobilized and then laparoscope was removed.
  11. The midline incision was extended over left peri-umbilical area.
  12. The ascending colon was delivered into the incision and points of transection were selected proximally and distally.
  13. 15-20Ccm away from Ileocecal valve of samll bowel was decided and transected, anvil 29mm was inserted in to the small bowel luemn and purstring suture was made by purstring clamp.
  14. The determined transected T colon was selected, a incision was made and EEA deviced was inserted and a end-to side anastomisis was made. The T colon stump was stapled with Open GIA 75mm. Interrupted 3-0 silk Lambert sutures were used to re-inforcement at outer layer
  14. The anastomosis was checked and found to be intact and widely patent.
  15. The small bowel and colon was put back into the abdomen. Pneumoperitoneum was established again.
  16 We consult GU doctor to perform removal of right ureter stone and D-J insertion.
  16. Possible bleeder was examed again. The orientation of the small bowel was checked thoroughly.
  17. The fascia was closed with interrupted sutures via Num.1 Vicryl. The fascia of the 12 mm trochar wound was closed with 1-0 Vicryl.
  18. The skin wound was sutured with 3-0 Nylon and then cover with Medipore.
  19. The patient tolerated the procedure well and was brought to the POR in stable condition.
- 器械數量: 正確
- 紗布數量: 正確

Image

- PAINT
  - abdomen

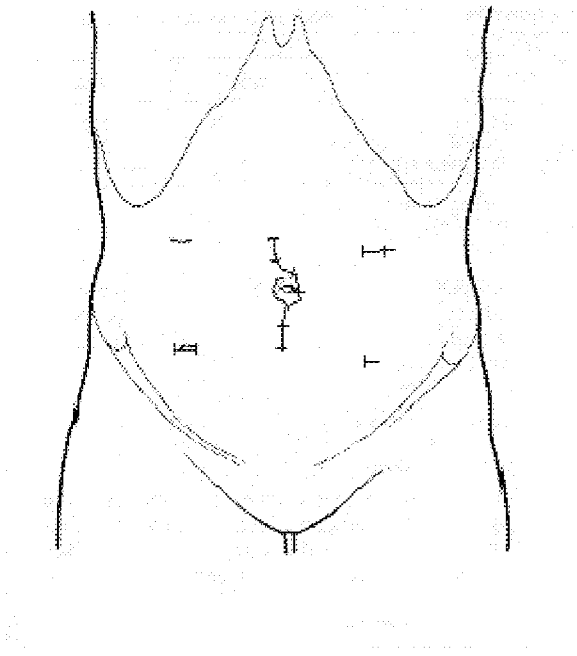

  - colon & rectum

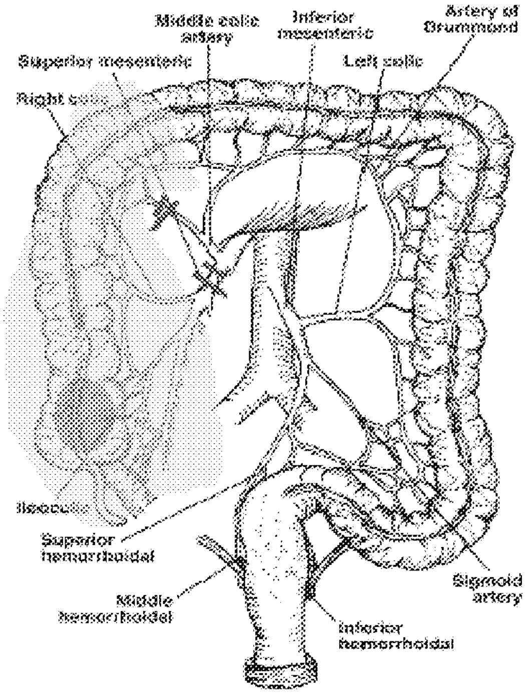

主任 2062F蔣鋒帆 / 醫師 2332G林志安

正式摘要(電子簽章：000000233752416)

W52 / W52

doctorRank:B
